# Nanoparticle T cell engagers for the treatment of acute myeloid leukemia

**DOI:** 10.18632/oncotarget.28054

**Published:** 2021-09-14

**Authors:** Kinan Alhallak, Jennifer Sun, Barbara Muz, Amanda Jeske, Jessica Yavner, Hannah Bash, Chaelee Park, Berit Lubben, Ola Adebayo, Samuel Achilefu, John F. DiPersio, Abdel Kareem Azab

**Affiliations:** ^1^Department of Radiation Oncology, Washington University School of Medicine, St. Louis, MO 63110, USA; ^2^Department of Biomedical Engineering, Washington University McKelvey School of Engineering, St. Louis, MO 63130, USA; ^3^Department of Radiology, Washington University School of Medicine, St. Louis, MO 63110, USA; ^4^Department of Medicine, Washington University School of Medicine, St. Louis, MO 63110, USA

**Keywords:** acute myeloid leukemia, T cell engagers, nanoparticles, 3D tissue culture model

## Abstract

Acute myeloid leukemia (AML) is the most common type of leukemia and has a 5-year survival rate of 25%. The standard-of-care for AML has not changed in the past few decades. Promising immunotherapy options are being developed for the treatment of AML; yet, these regimens require highly laborious and sophisticated techniques. We create nanoTCEs using liposomes conjugated to monoclonal antibodies to enable specific binding. We also recreate the bone marrow niche using our 3D culture system and use immunocompromised mice to enable use of human AML and T cells with nanoTCEs. We show that CD33 is ubiquitously present on AML cells. The CD33 nanoTCEs bind preferentially to AML cells compared to Isotype. We show that nanoTCEs effectively activate T cells and induce AML killing *in vitro* and *in vivo*. Our findings suggest that our nanoTCE technology is a novel and promising immuno-therapy for the treatment of AML and provides a basis for supplemental investigations for the validation of using nanoTCEs in larger animals and patients.

## INTRODUCTION

Acute myeloid leukemia (AML) is the most common type of leukemia; it is characterized by the overproduction of immature myeloid stem cells in the bone marrow and has a 5-year survival rate of around 25% [1, 2]. The survival curves for AML patients have remained stagnant in the past decades due to the lack of newly approved therapies for AML. However, recent development in novel therapeutics and technologies have shown promising results in preclinical and clinical settings [3–5].

Exciting immunotherapy technologies that are being investigated for AML include chimeric antigen receptor T (CAR-T) cells and bispecific T cell engagers (TCEs). CAR-T cells are autologous T cells that have been virally transfected to express an engineered CAR construct, containing a synthesized fragment that targets the desired surface antigen on the target cell [6]. However, the main disadvantages of this technology relative to traditional therapies include toxicity, the long-term safety profile of the viral vector, the need for frequent quality control testing throughout production, the high costs due to the need of extensive labor and expensive facility, complex production, and the inability to target multiple tumor antigens with one CAR-T cell [7, 8].

In addition to CAR-T cells, T cell-based therapy can be pursued with TCEs. TCE consists of two single chain variable fragments which are connected by a protein linker. One of the domains recognizes a tumor-associated surface antigen, while the other recognizes the T cell CD3 receptor [9]. This enables the TCE to redirect the T cell to the tumor and induce subsequent activation and expansion of the T cell. TCEs stimulate endogenous T cells and demonstrate high potency and efficacy against tumor cells [10–12], circumventing certain limitations of genetically engineering extracted patient T cells to express CARs. This immunotherapeutic option has been shown to be successful for both solid and liquid tumors, but is mostly known for the treatment of hematological malignancies [13]. The disadvantages of TCEs, however, include toxicity, expensive costs for labor and production, complex production, poor pharmacokinetics profile (t_1/2_ ~ 2 hours), and the inability to target multiple cancer surface markers [10, 14, 15].

We have previously developed a nanoparticle-based T cell engagers (nanoTCEs) technology that is based on conjugation of two monoclonal antibodies (mAbs) to the surface of a liposomal nanoparticle; one antibody is against a cancer antigen and the other is against the CD3 receptor on T cells [16]. NanoTCEs utilize existing mAbs which we conjugate to the surface of a nanoparticle, therefore taking advantage of the high specificity of existing mAb-based therapies, to engage and direct robust responses from the immune system (T cells). NanoTCEs have been shown to have clear advantages compared to both CAR-T cells and TCEs; nanoTCEs are: 1) simple to produce – the production of nanoparticles and chemical conjugation of readily available mAbs takes only a few hours; 2) prolonged pharmacokinetic profile (t_1/2_ ~ 60 hours), 3) modular platform allowing customizable targeting of multiple tumor and immune cell antigens [16]. Moreover, nanoTCEs has demonstrated therapeutic efficacy in endogenous T cell activation as well as T cell-directed cancer cell lysis, both *in vitro* and *in vivo*. Therefore, the nanoTCE technology represents a facile platform for development of T-cell engagement immunotherapy using any existing anti-cancer mAbs.

There is a long-standing interest in CD33-targeted therapies AML. CD33 is a myeloid-associated marker found mainly on cells committed to the myeloid lineage and its expression is absent on non-hematopoietic cells [17]. High CD33 expression has been reported on AML blasts; data show as much as 85–90% of blasts express CD33 in AML patients [18, 19]. Moreover, CD33 expression positively correlates with stage of the disease [2]. Several therapy options using CD33 as a target have been under development pre-clinically and clinically. The CD33-directed antibody-drug conjugate, gemtuzumab ozogamicin is FDA approved for AML [20]. Studies on CD33-targeted TCEs for treatment of AML have also demonstrated efficacy potential. CD33 x CD3 bi-specific TCE, AMG 330, is currently in Phase I clinical trial for relapsed/refractory as well as minimal residual disease positive AML (NCT02520427) [21–23]. Additionally, CD33 x CD3 tandom diabody, AMV564, is under Phase I clinical trial for relapsed/refractory AML (NCT03144245) [24, 25]. CD33-targeted CAR-T cells have also been explored and proven effective [26, 27]; CD33-CART cells are being explored in multiple clinical trials for children and adults with relapsed/refractory AML (NCT03971799, NCT03927261). Thus, a plethora of evidence validates CD33 as a targetable biomarker for immunotherapy in AML.

In this study, we sought to create a nanoTCE targeted to CD33 for the treatment of AML, as a versatile T-cell engagement platform.

## RESULTS

**Figure 1:**
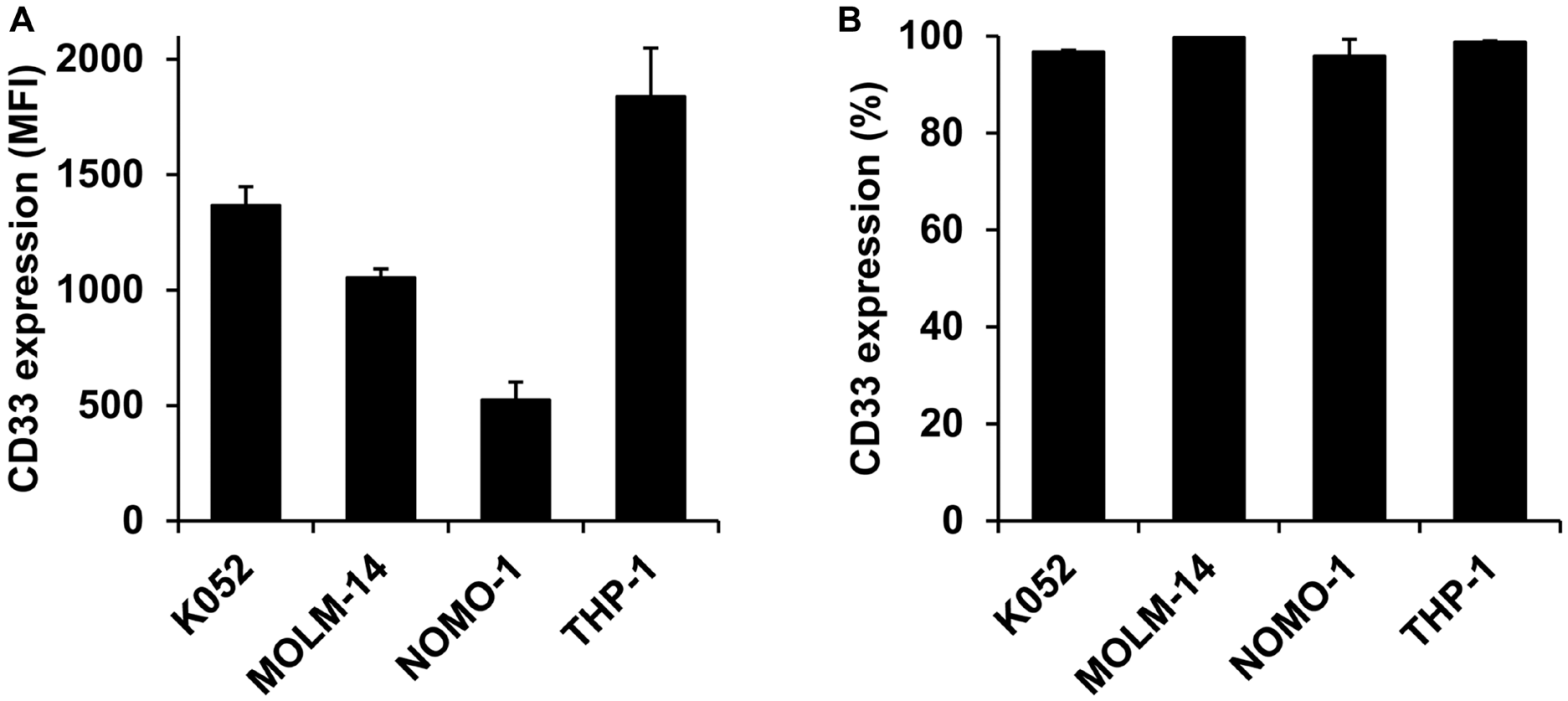
CD33 expression on AML cell lines. (**A**) Mean fluorescent intensity and (**B**) percent expression on K052, MOLM-14, NOMO-1, and THP-1. Data is represented as mean ± standard deviation.

CD33 is a valuable target for the treatment of AML, therefore, we first validated the presence of the marker in our experimental setup. We measured the fluorescent intensity and percent of CD33 in four different human AML cell lines. For all cell lines, CD33 was expressed in high levels (Figure 1A) and uniformly on 90–100% of the cells (Figure 1B), indicating that CD33 is a promising surface marker for nanoTCE targeting.

**Figure 2:**
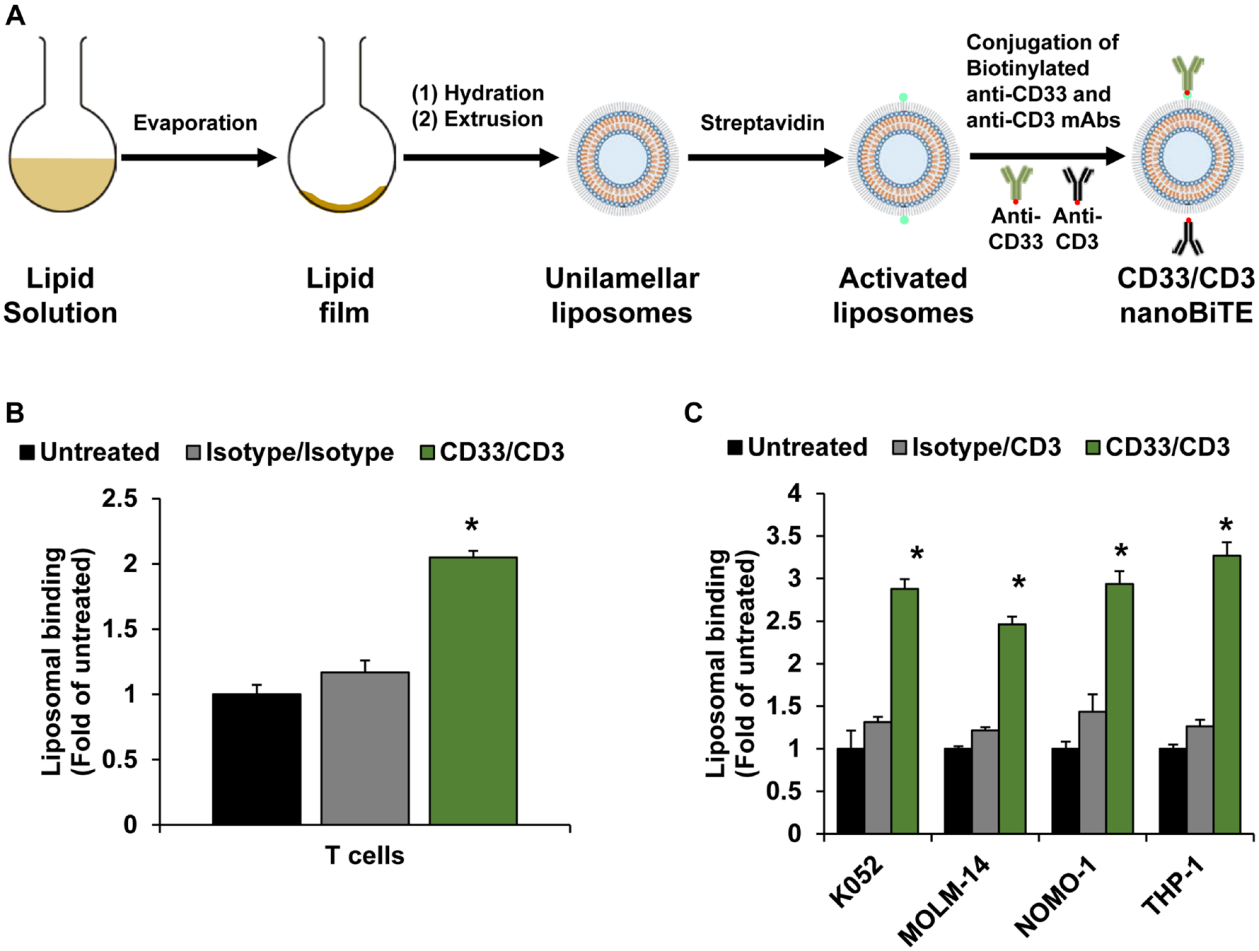
Development of nanoTCEs for AML. (**A**) Schematic of CD33/CD3 nanoTCEs production process. (**B**) Liposomal binding of Isotype and CD33/CD3 nanoTCEs to T cells. (**C**) Liposomal binding of Isotype and CD33/CD3 nanoTCEs to AML cell lines. Statistical significance between CD33/CD3 and Isotype is indicated by ^*^(*p* < 0.05). Data is represented as mean ± standard deviation.

Next, we developed AML-targeting CD33/CD3 nanoTCEs and prepared Isotype/CD3 nanoTCEs as control. A schematic of the liposomal CD33/CD3 nanoTCE production process is shown in Figure 2A. We characterized the physicochemical properties of these nanoTCEs, including diameter, polydispersity index (PDI), and zeta potential which are shown in Table 1. We found that the properties are in accord with our previous report [16], in which the size of the nanoTCEs was about 140 nm, with low PDI indicating the uniformity of the particle size, and with close to neutral net charge.

**Table 1:** Characterization of nanoTCEs^1^

Formulation	Diameter (nm)	PDI^2^	Zeta Potential (mV)
Isotype/CD3	140.5 ± 1.0	0.11 ± 0.02	0.9 ± 0.2
CD33/CD3	141.3 ± 1.0	0.07 ± 0.01	1.1 ± 0.1

^1^Mean ± standard deviation; ^2^polydispersity index (PDI).

We then tested the binding of CD33/CD3 nanoTCEs to T cells and AML cell lines compared to Isotype controls. The CD33/CD3 nanoTCEs bound to T cells at 2-fold higher than Isotype (Figure 2B), and bound to AML cell lines around 2- to 3-fold higher compared to Isotype (Figure 2C).

**Figure 3:**
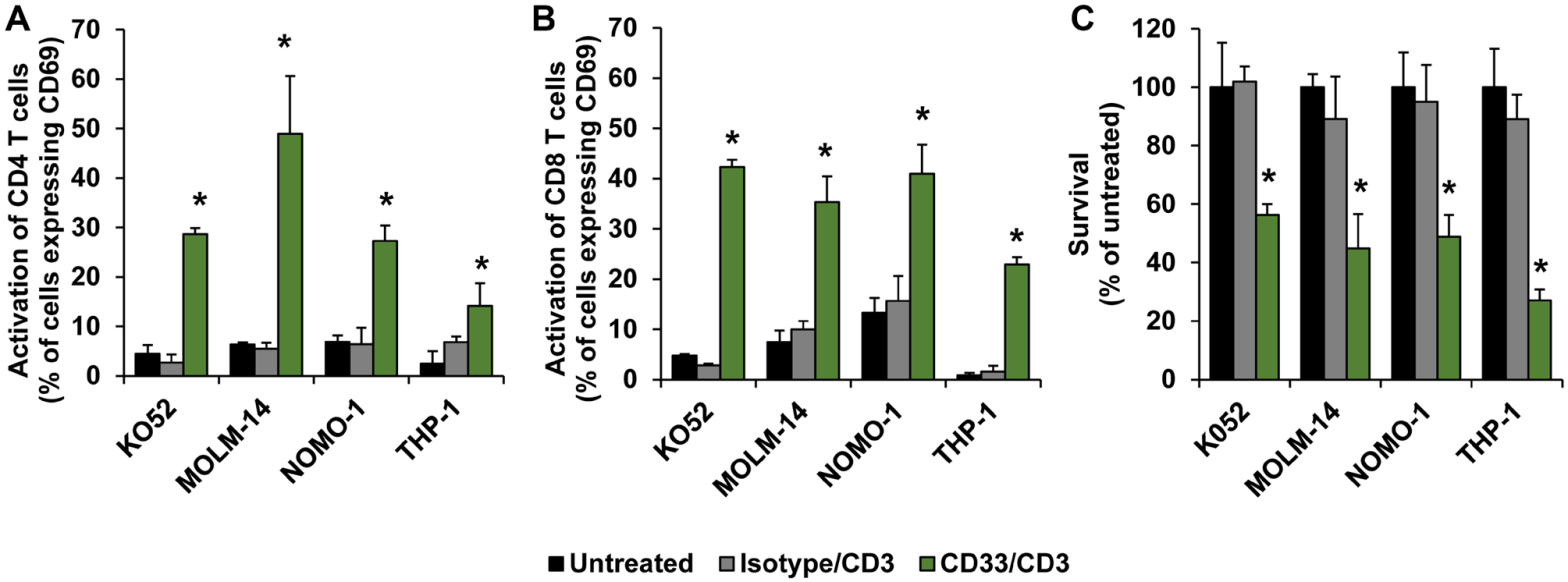
Efficacy of nanoTCEs *in vitro*. The effect of Isotype/CD3 and CD33/CD3 nanoTCEs treatment on activation of (**A**) CD4 and (**B**) CD8 T cells, and on (**C**) survival of AML cell lines, in 3DTEBM after 4 days. Statistical significance between CD33/CD3 and Isotype is indicated by ^*^(*p* < 0.05). Data is represented as mean ± standard deviation.

To demonstrate the therapeutic efficacy of nanoTCEs *in vitro*, we investigated the effect of nanoTCEs on activation of T cells as well as T cell-mediated killing of AML cell lines in our 3DTEBM culture model [28]. 3DTEBM is a patient-derived 3D cell culture system that mimics the leukemic bone marrow niche, in which it recapitulates the tumor microenvironment and drug resistance superior than classic 2D cultures [29]. Activation of T cells was observed as increase in CD69 upregulation in CD4 (Figure 3A) and CD8 T cells (Figure 3B) following co-culture of T cells with AML cell lines with CD33/CD3 nanoTCEs, but not with Isotype/CD3 TCEs. We have shown previously that the nanoTCE is not able to activate T cells alone; this is shown by the use of the Isotype/CD3. T cells do not activate following the binding of the nanoTCE alone; it only works following the engagement of the T cell and the target cell via nanoTCE which aligns with the kinetic segregation model for T cell receptor triggering [16, 30]. Consequently, no T cell-mediated killing of AML cells was observed following treatment with Isotype/CD3 TCEs, while 50–75% killing was observed following treatment with CD33/CD3 nanoTCEs for 4 days (Figure 3C).

**Figure 4:**
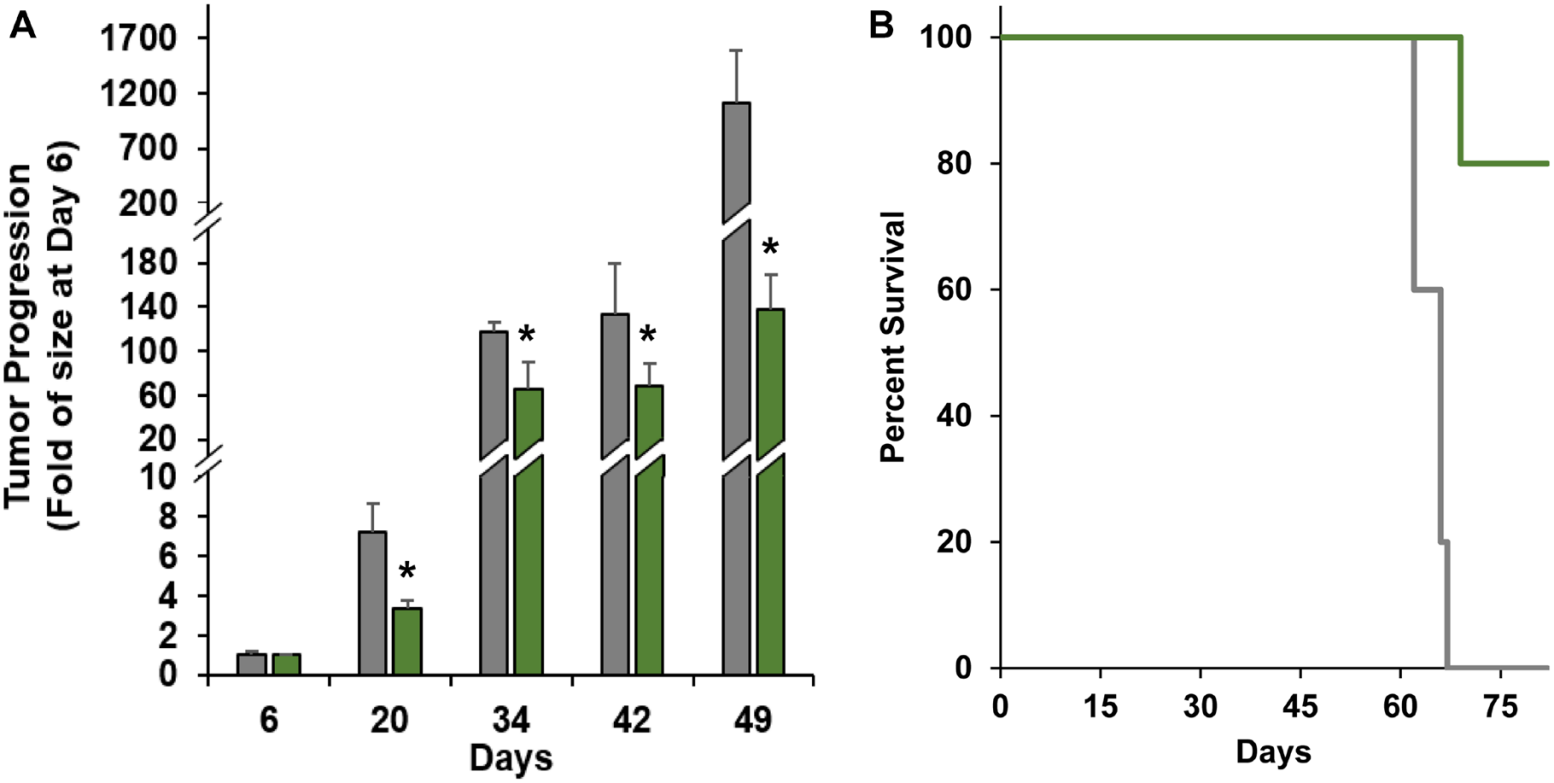
Efficacy of nanoTCEs *in vivo*. (**A**) Tumor progression and (**B**) Kaplan-Meier survival comparison between mice treated with Isotype/CD3 (grey; *n* = 5) or CD33/CD3 nanoTCEs (green; *n* = 5). Statistical significance between CD33/CD3 and Isotype is indicated by ^*^(*p* < 0.05). Tumor progression data is represented as mean ± standard deviation for tumor progression.

To demonstrate the therapeutic efficacy of nanoTCEs *in vivo*, we injected human AML THP-1 cells genetically engineered to express luciferase in an NCG immunocompromised mice model. At Day 7 of tumor inoculation, we injected human primary T cells to the mice and treated with nanoTCEs weekly thereafter. Mice treated with CD33/CD3 nanoTCEs had significantly lower tumor burden at all time points compared with Isotype/CD3 nanoTCEs (excluding Day 6) (Figure 4A). Additionally, 100% of the CD33/CD3 cohort was alive at the end of the study, while only 40% of Isotype/CD3 cohort survived past Day 62 and none past Day 66 (Figure 4B).

## DISCUSSION

AML is associated with low survival rates and novel therapeutics are direly in need. In this study, we validated that CD33 is an abundant and relevant marker on AML cells, and demonstrated that our CD33/CD3 nanoTCE technology can induce T-cell directed cytotoxic activity against AML. The CD33/CD3 nanoTCEs bound preferentially to AML and T cells; this enables specific binding to only these cells and prevents binding to other hematopoietic cells to reduce off-target toxicities. T cell activation and T cell-mediated AML cell lysis was induced following the use of the nanoTCEs *in vitro* and *in vivo*.

Our nanoTCE platform uses nanoparticles to create a relatively simple to produce, reproducible, and off-the-shelf solution to overcome the major limitations associated with current immunotherapy techniques such as TCEs and CAR-T cells. Importantly, this technology is highly customizable and provides the flexibility to engage any immune cell for the treatment of the cancer of interest. In this study, we report a CD33/CD3 nanoTCE that targets the CD33 antigen with high specificity using mAbs, which enables a potent and efficacious immunotherapy treatment against AML. Future studies are warranted to investigate this therapy in combination with chemotherapy, the extent this technology eliminates minimal residual disease and relapse, as well as its efficacy in AML patients.

## MATERIALS AND METHODS

### Materials and reagents

Antibodies and Pan T Cell Isolation Kits were purchased from Miltenyi Biotec (Bergisch Gladbach, Germany). RPMI-1640, 0.25% trypsin, L-glutamine, and penicillin-streptomycin were purchased from Corning (Corning, NY). Fetal bovine serum, lipophilic tracers, collagenase, and counting beads were purchased from Life Technologies (Carlsbad, CA). 1,2-dipalmitoyl-sn- glycero-3-phosphocholine (DPPC), 1,2-distearoyl-sn-glycero-3-phosphoethanolamine-N- [amino(polyethylene glycol)-2000] (DSPE-PEG2000), and extrusion membranes were purchased from Avanti Polar Lipids (Alabaster, AL). Cholesterol was purchased from Millipore Sigma (Burlington, MA). Streptavidin conjugation kit was purchased from Abcam (Cambridge, United Kingdom). Lipophilic tracer DiO was purchased from Invitrogen (Eugene, OR).

### Cell culture and 3DTEBM

K052, MOLM-14, NOMO-1, and THP-1 cell lines were all obtained from the lab of John DiPersio. Peripheral blood mononuclear cells (PBMCs) were isolated from healthy donors using Ficoll-Paque PREMIUM (Millipore Sigma), and T cells were separated using a Pan T cell isolation kit. Cell lines were cultured in RPMI-1640 supplemented with 10% fetal bovine serum, 2 mM of L-glutamine, and 1% penicillin-streptomycin. All cell cultures were cultured in NuAire water jacket incubators (NuAire, Plymouth, MN) at 37°C and in 5% CO_2_.

3D tissue engineered bone marrow (3DTEBM) cultures were established in 96-well plates by cross-linking fibrinogen in patient bone marrow supernatant at a concentration of 1 and 4 mg/mL calcium chloride and tranexamic acid [28]. The 3DTEBM was supplemented with media on top after gelling. At time of analysis, cells were retrieved by digesting 3D scaffolds with collagenase (Gibco, Life Technologies) for 2 h at 37°C.

### Preparation and characterization of nanoTCEs

The procedure of making nanoTCEs has been previously described [16]. Briefly, nanoTCEs were prepared with three components: cholesterol, DPPC, and DSPE-PEG2000 with a molar ratio equivalent to 30: 65: 5. Lipids were mixed and solubilized in chloroform, and evaporated through a rotary evaporator (Heidolph, Schwabach, Germany) to form a thin lipid film. The film was then hydrated, and the resulting suspension was extruded using the Avestin LiposoFast LF-50 (Ottawa, ON, Canada) with 100 nm polycarbonate membranes [31, 32]. The biotinylated antibodies (Isotype, CD3, and/or CD33) were conjugated to the liposomes using streptavidin and biotin reaction [33]. Malvern Zetasizer Nano ZS90 (Malvern, Worcestershire, United Kingdom) was used to determine zeta potential, diameter, and polydispersity index. Fluorescent liposomes were prepared by dissolving DiO in the lipid/chloroform mixture before film formation.

### Protein expression

Cells were stained with anti-CD33 APC antibody in 4°C for one hour, washed, and analyzed by flow cytometry using MACSQuant Analyzer 10 (Miltenyi Biotec) with an Ex/Em of 635/655–730 nm. Cells were gated using forward and side scatter and analyzed for relative mean fluorescent intensity (MFI) of APC using BD FlowJo Software [34, 35].

### Liposomal binding

Each nanoTCE were prepared with a lipophilic fluorescent tracer DiO. Cell lines and T cells were treated with Isotype/Isotype or CD33/CD3 nanoTCEs (3.7 nM) for two hours at 37°C. Cells were spun down, washed, and analyzed by flow cytometry with Ex/Em of 488/525 ± 25 nm. Cells were gated using forward and side scatter and analyzed for MFI of DiO using BD FlowJo Software.

### Activation of T cells and T cell-mediated killing of AML *in vitro*

Cell lines were cultured with healthy donor T cells in the 3DTEBM and treated with Isotype/CD3 or CD33/CD3 nanoTCEs at a concentration of 3.7 nM for 4 days. Cultures were digested, and cells were retrieved and stained with anti-CD3 PE, anti-CD4 FITC, anti-CD8 Violet, and anti-CD69 APC antibodies for one hour at 4°C. Samples were analyzed by flow cytometer with Ex/Em of 488/585 ± 20, 488/525 ± 25, 405/450 ± 25, and 635/655-730 nm, respectively. Cells were gated using forward and side scatter followed by double positive CD3+/CD4+ or CD3+/CD8+, both of which were analyzed for % of cells positive for CD69 using BD FlowJo Software.

### T cell-mediated Killing of AML *in vitro*

Cell lines (pre-labeled with DiO) were incubated with healthy donor T cells in the 3DTEBM and treated with Isotype/CD3 or CD33/CD3 nanoTCEs at a concentration of 3.7 nM for 4 days. Counting beads were added to the culture before matrix digestion. The cells were retrieved and analyzed by flow cytometry. Number of AML cells were analyzed as DiO+ cells and normalized to the number of counting beads using BD FlowJo Software.

### T cell-mediated Killing of AML *in vivo*

Immunodeficient NCG mice (strain: 572), female, 50–56 days old, were purchased from Charles River (Wilmington, MA), and all experiments using these rodents were in compliance with the Institutional Animal Care and Use Committee at Washington University. Human AML cell line, THP-1 CBR cells (1 × 10^6^/mouse) were injected intravenously (i.v.) into 10 NCG mice. One week after tumor inoculation, human T cells (5 × 10^6^/mouse) were injected i.v. Mice were randomized into two groups (*n* = 5) and were treated i.v. with Isotype/CD3 or CD33/CD3 nanoTCEs (0.5 mg/mouse) weekly for a total of four weeks. Tumor progression was tracked by weekly bioluminescent imaging. Mice were injected with D-luciferin (150 μg/kg) intraperitoneally, and tumor burden was detected using an IVIS 50 bioluminescence imaging system (PerkinElmer, Waltham, MA, USA) 10 minutes post-luciferin injection, and images were analyzed using Living Image 2.6 software (PerkinElmer). Mice were monitored on a daily basis to record survival.

### Statistical analyses

All experiments were independently replicated three times and performed in quadruplicates, and animal experiments consisted of five mice per group; data from *in vitro* and *in vivo* experiments were expressed as means ± standard deviation. Statistical significance was analyzed using a Student’s *t*-test, one-way, or two-way analysis of variance. Log-rank test was used to compare the Kaplan Meier curves. *P*-values less than 0.05 were used to indicate statistically significant differences.

## Abbreviations

AML: Acute myeloid leukemia
CAR-T cells: chimeric antigen receptor T cells
TCEs: bispecific T cell engagers
nanoTCEs: Nanoparticle T cell engagers
mAbs: monoclonal antibodies
3DTEBM: 3D tissue engineered bone marrow
